# Many Options, Few Solutions: Over 60 My Snakes Converged on a Few Optimal Venom Formulations

**DOI:** 10.1093/molbev/msz125

**Published:** 2019-05-20

**Authors:** Agneesh Barua, Alexander S Mikheyev

**Affiliations:** 1 Okinawa Institute of Science and Technology Graduate University, Onna, Japan; 2 Evolutionary Genomics Research Group, Ecology and Evolution Unit, Australian National University, Canberra, Australia

**Keywords:** gene expression, generalized linear mixed model, macroevolution, parallel evolution, venom

## Abstract

Gene expression changes contribute to complex trait variations in both individuals and populations. However, the evolution of gene expression underlying complex traits over macroevolutionary timescales remains poorly understood. Snake venoms are proteinaceous cocktails where the expression of each toxin can be quantified and mapped to a distinct genomic locus and traced for millions of years. Using a phylogenetic generalized linear mixed model, we analyzed expression data of toxin genes from 52 snake species spanning the 3 venomous snake families and estimated phylogenetic covariance, which acts as a measure of evolutionary constraint. We find that evolution of toxin combinations is not constrained. However, although all combinations are in principle possible, the actual dimensionality of phylomorphic space is low, with envenomation strategies focused around only four major toxin families: metalloproteases, three-finger toxins, serine proteases, and phospholipases A2. Although most extant snakes prioritize either a single or a combination of major toxin families, they are repeatedly recruited and lost. We find that over macroevolutionary timescales, the venom phenotypes were not shaped by phylogenetic constraints, which include important microevolutionary constraints such as epistasis and pleiotropy, but more likely by ecological filtering that permits a small number of optimal solutions. As a result, phenotypic optima were repeatedly attained by distantly related species. These results indicate that venoms evolve by selection on biochemistry of prey envenomation, which permit diversity through parallelism, and impose strong limits, since only a few of the theoretically possible strategies seem to work well and are observed in extant snakes.

## Introduction

Single genes underlying major traits are the exception rather than the rule, and the dissection of polygenic trait variation has been at the forefront of biological research (Lander and Kruglyak 1995; Nadeau 2001; Morley et al. 2004). Much of the complexity resulting from interactions between genes is mediated through their expression, which plays a central role in determining phenotypic variation between individuals and populations (Deutsch et al. 2005; Cardoen et al. 2011; de Montaigu et al. 2015; Ghalambor et al. 2015; Catalán et al. 2016). In particular, levels of gene expression account for substantial sources of variation in natural populations, acting as potential targets of natural selection (Oleksiak et al. 2002; Deutsch et al. 2005; Harrison et al. 2012). Although population-level differences in expression may contribute to the onset of local adaptation and perhaps even eventual adaptive divergence (Nolte et al. 2009; Jeukens et al. 2010; Ghalambor et al. 2015), how changes in gene expression levels lead to evolution of complex traits over the course of millions of years remains largely unknown.

Interactions between genes and their effect in channeling of adaptive responses have been the focus of the field of quantitative genetics. How evolution results from the combined effects of the adaptive landscape, and the pattern of genetic variances and covariance among genes (the *G* matrix), is one of the key questions in this field (Lande 1979; Arnold et al. 2008). The covariance between genes plays a vital role in shaping complex traits by determining the evolutionary trajectory through natural selection (Arnold et al. 2001), and the occurrence of parallelism (Rosenblum et al. 2014). Although most quantitative genetics studies deal with populations, their conclusions can translate to macroevolutionary processes as well. For example, estimates of divergence between populations show that the direction of greatest phenotypic divergence can be predicted by the multivariate direction of greatest additive genetic variance within populations (Schluter 1996). Unfortunately, the *G* matrix cannot be extrapolated across macroevolutionary timescales, as it itself evolves (Steppan et al. 2002). Fortunately, it is possible to compute a phylogenetic covariance (PCOV) matrix for multivariate traits, which can serve as a useful analogy to the *G* matrix, but over much larger timescales, and incorporating a broader range of constraints (Lynch 1991; Adams and Felice 2014). We can then examine whether the structure of the PCOV matrix corresponds to evolutionary trajectories of complex traits.

Here, we use the analogy between the *G* matrix and the PCOV matrix to understand how gene expression evolves in a complex trait, namely snake venom. Being composed of proteinaceous cocktails, snake venoms are unique in that the expression of each toxin type can be quantified and traced to a distinct genomic locus (Rokyta et al. 2012, 2013; Aird et al. 2017; Margres, Bigelow, et al. 2017; Shibata et al. 2018). Variations in gene expression alter the abundance of proteins in the venom, thereby influencing venom efficacy (Daltry et al. 1996; Gibbs and Mackessy 2009; Casewell et al. 2011; Holding et al. 2016; Margres, Wray, et al. 2017). Thus, toxin expression levels constitute the polygenic phenotype that is the venom, allowing us to examine how selection affects gene expression over tens of millions of years. To examine the features of complex trait evolution at the level of gene expression, we estimated phylogenetic covariance of 10 toxins families using data from 52 snake species covering the 3 venomous snake families (Elapidae, Viperidae, and Colubridae) and asked the extent to which our observed patterns corroborate already known instances of evolutionary change across taxa.

Although we find that extant snake venoms occupy a limited area of phenotypic space, largely centered on four major toxin families, we find no evidence of phylogenetic constraints on the number of possible venom combinations. These data show that the relatively small number of molecular strategies used by the snakes result from consistent and often convergent selection on the biochemistry of envenomation, rather than from intrinsic constraints on gene interactions. Thus, over tens of millions of years selection likely plays a greater role in shaping the venom phenotype than intrinsic constraints.

## Results

### Expression Data and Phylogeny

Expression data for snakes were collected from published studies that reported relative levels of toxin expression via next-generation transcriptome sequencing of cDNA libraries. We obtained data for a total of 52 different snake species from the 3 major venomous families (Colubridae, Elapidae, and Viperidae), from a list of 39 publications (supplementary table 1, Supplementary Material online). For inclusion, each study had to provide quantitative data on toxin component abundance and had species for which phylogenetic data were available. We restricted our data set to include components that are found in at least 50% of snakes (supplementary fig. 35, Supplementary Material online). We focused on generally important toxin families, because sample sizes for the other components would be too low for accurate and phylogenetically unbiased inference, an approach similar to that of Junqueira-de-Azevedo et al. (2016). Incidentally, this cut-off also eliminated many low-abundance toxin families (on average <1% of the venom, supplementary fig. 34, Supplementary Material online). The abundance of these toxins would be more difficult to estimate, as they are closer to the signal to noise to threshold of gene expression experiments. Overall 10 out of 25 toxin families we retained. For comparative analyses, we used a published time-calibrated phylogeny of squamates, which estimated the most recent common ancestor (root) of the three snake families to about 60 Ma (Zheng and Wiens 2016).

### Evolutionary Covariance between Venom Components

**Fig. 1.**
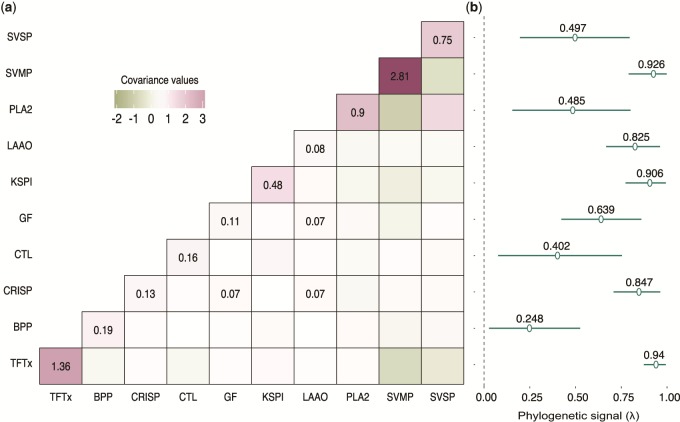
Phylogenetic constraints on individual toxins and their combinations. (*a*) A lack of significant values (only significant values labeled) in the PCOV matrix denotes a lack of phylogenetic constraint between toxin families. (*b*) Components show a significant presence of a phylogenetic signal, indicating that closely related species are likely to evolve the same way. Lambda, represents phylogenetic signal, which is a measure of dependency of trait evolution with phylogeny. Lambda values, are estimated as toxin variance on the diagonal, divided by the sum of diagonal variance and residuals. TFTx, SVMP, KSPI, LAAO, and CRISP showed the highest signal, with greatest significance, whereas the rest showed comparatively weaker signals. Phylogenetic constraints determine convergence and parallel evolution, where high constraint reduces the likelihood of genes contributing to different convergent regimes (Rosenblum et al. 2014). Yet, for snake venom genes, we see no such constraints in gene expression despite the high phylogenetic signal, suggesting that all toxin combinations, in principle, are possible.

By limiting the range of responses to natural selection, the covariances between genes reflect constraints that shape a phenotype. The PCOV matrix accounts for the effect of phylogeny on the interrelationships between genes coding for the snake venom phenotype, providing an approximation of the presence or absence of constraint behind the evolution of gene expression levels. To estimate the PCOV, we used a phylogenetic generalized linear mixed model (PGLMM) under a Bayesian framework. The concept of PGLMM was devised in the early 90s as a method to infer evolutionary constraints of characters using only phylogeny and measures of phenotypes and is based on the animal model in quantitative genetics (see Materials and Methods) (Lynch 1991; Wilson et al. 2010). As an extension of maximum likelihood–based techniques like phylogenetic least squares, PGLMM was notable for its versatility as a comparative method (Miles and Dunham 1993; de Villemereuil and Shinichi Nakagawa 2014). We use a modern rendition of the PGLMM devised by Hadfield and Nakagawa, which was optimized for faster and better performance (Hadfield and Nakagawa 2010; de Villemereuil and Shinichi Nakagawa 2014). The mean effective sample size for all parameters was greater than 11,000 (supplementary fig. 4, Supplementary Material online). The diagnostics revealed suitable convergence of the chains with negligible autocorrelation in the Markov chain Monte Carlo (MCMC; supplementary figs. 1–3, Supplementary Material online). Significant values in the PCOV matrix denote the presence of phylogenetic constraint, whereas nonsignificant values denote its absence. We observed a lack of significant values in the PCOV (fig. 1) for all the venom components that we modeled. In addition to estimating a PCOV, the model was used to compute *λ* values which denote the phylogenetic signal (fig. 1). Phylogenetic heritability of a trait is defined as the proportion of variance explained by the relationship among species given by the phylogeny, and in our case it is equivalent to Pagel’s lambda model of phylogenetic signal which is similar to that of Lynch’s original phylogenetic heritability (Freckleton et al. 2002; Housworth et al. 2004; de Villemereuil and Shinichi Nakagawa 2014). The *λ* values are a measure of statistical dependence of trait values and phylogeny. They indicate whether certain components in modern snakes were likely similar as in their ancestors. In our case, most venom components show strong phylogenetic signals of greater than 0.5, albeit with large confidence intervals. However, all venom components have *λ* significantly greater than 0. A few, in particular cysteine-rich secretory proteins (CRISPs), snake venom metalloproteinase (SVMP), three-finger toxins (TFTx), and Kunitz-type serine protease inhibitor (KSPI), show very strong phylogenetic signals (>0.8) and narrow confidence intervals, indicating the presence of strong phylogenetic inertia.

#### Compositional Data Considerations

It should be noted that the main analyses were performed on compositional (sum-constrained) data, which has the potential of introducing spurious correlations. A range of common solutions to this problem involve log-transformations of the data (Aitchison and Egozcue 2005), which allows for the comparison of relative quantities of the components. However, structural zeros cannot undergo log-transformations, but also cannot be excluded from a comparative analysis because they represent biologically valid characters. Nonetheless, we validated the robustness of the main results using the centered-log-ratio (clr) transform using the *compositions* R package (van den Boogaart and Tolosana-Delgado 2008) and imputed zero values in our compositional data using the “cmultRepl” function in the *zCompositons* R package (Palarea-Albaladejo and Martín-Fernández 2015), to confirm that the overall structure of the covariance matrix is unchanged. Indeed, although the PGLMM using transformed data had significantly worse fit, we did not detect more off-diagonal correlations, and the on-diagonal values were still high (supplementary fig. 37, Supplementary Material online).

### Four Toxin Families Drive the Evolution of the Snake Venom Arsenal

**Fig. 2.**
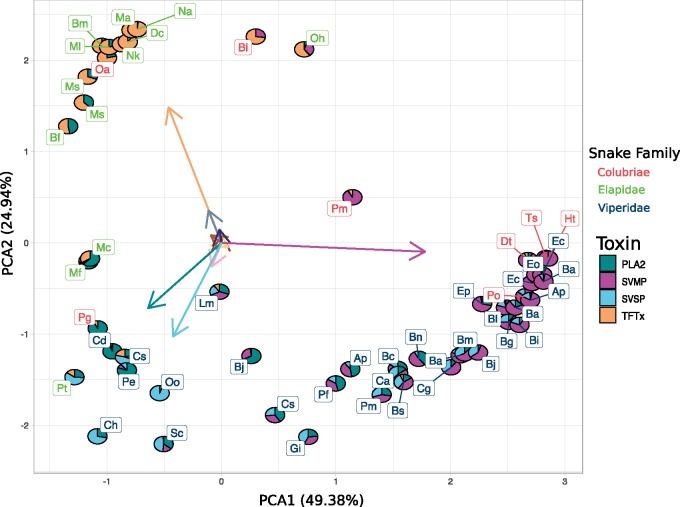
Snakes (species codes provided in Supplementary Material online) cluster on phylomorphospace along the axes of four toxins: PLA2, SVSPs, SVMPs, and TFTx. These axes represent three distinct envenomation strategies employed by the snakes. Vipers in our data employ a wide spectrum of strategies, from being focussed primarily on SVMP, to employing a mixture of PLA2 and SVSP. Most elapids in our data employ a strategy primarily based on TFTx, whereas two Micrurus species (Mc, Mf) have a combination of PLA2 and TFTx. Colubrids show a unique trend of being scattered throughout the phylomorphospace, having at least one species adopting each of the three strategies. Despite the lack of constraint in gene expression, the snake venom phenotype has very low dimensionality with the four major components accounting for 74.3% of the variation. Clustering of distantly related snakes to around a similar strategy hints at the likely parallelism of these major toxins.

The PCOV is a measure of additive phylogenetic covariance, that can be used to estimate the direction of greatest adaptive phenotypic variation (Schluter 1996; Wilson et al. 2010). We identified axes of maximum variations in the toxin components using Principal component analysis (PCA) on the phylogenetic covariances, using it to visualize the dimensionality of the venom phenotype (Uyeda et al. 2015). The venom phylomorphospace has very low dimensionality as the first two components jointly explained 74.3% of the variation. The largest loadings were from four families of toxins: TFTx, SVMP, phospholipase A2 (PLA2), and snake venom serine protease (SVSP) (fig. 2). We therefore classified them as “major” toxins, representing three largely distinct envenomation strategies focussed around SVMP, TFTx, and a combination of PLA2 and SVSP.

The clustering of snakes on this phylomorphic venom space shows a clear association between family and the major component in the venom. For example, most elapids venoms form a cluster dominated by TFTx, which is the principal family found in their venom. On the other hand, vipers occupy a larger region of phylomorphospace because some have venoms dominated by SVMP, whereas others use different combinations of SVMP, SVSP, and PLA2. Finally, colubrid venoms are the most diverse in composition, employing all of the different strategies. A key observation in the PCA is that some distantly related species cluster together around the same envenomation strategy, suggesting parallel evolution.

It is important to note that PLA2s in elapids (group I) and vipers (group II) are produced by different loci and have apparently evolved independently (Lynch 2007; Gibbs and Rossiter 2008; Vonk et al. 2013; Dowell et al. 2016; Shibata et al. 2018). In order to account for any underlying family-specific evolutionary trend, we conducted a parallel analysis by splitting PLA2 into elapid PLA2 and viperid PLA2 (supplementary fig. 5, Supplementary Material online). This analysis produced qualitatively the same results as the combined analysis, though the first two components of the PCA explained less variance (62.3% as opposed to 74.3%). In particular, loadings for both elapid and viperid PLA2 were oriented in the same direction (supplementary fig. 10, Supplementary Material online), indicating that the direction of variation in the phylomorphospace is the same for both groups. Thus, we carried out all subsequent analysis by combining them into a joint functional category.

### Parallelism of Envenomation Strategies

**Fig. 3.**
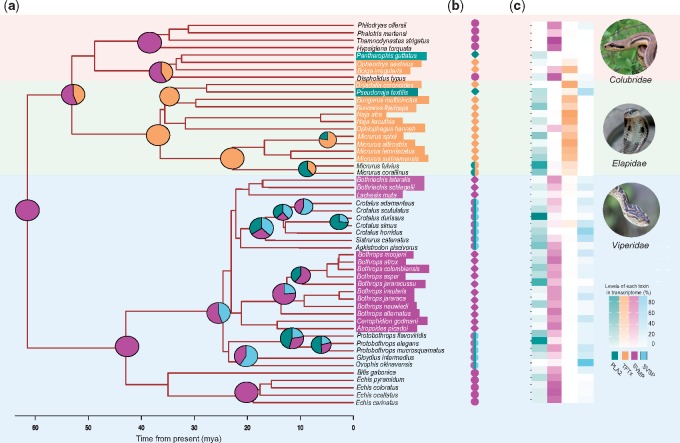
Evolution of the four major venom toxins and convergent phenotypic regimes in snakes. (*a*) Pie charts at selected nodes represent ASRs of the four major toxins (PLA2, TFTx, SVMP, and SVSP). For clarity, only the nodes where substantial changes in toxin levels took place are shown. Because snake venom composition has evolved dynamically, the ancestral venom (at the root 60 Ma) is difficult to estimate. Although only SVMP was reconstructed as present with a high degree of likelihood, albeit at low levels in our analysis, we would not rule out the presence of other venom components, particularly at low levels. For instance, SVSP does occur in all three families, though not detected at the root. Also, ancestral recruitment of a number of toxin compounds has been argued previously (Fry and Wüster 2004). Lineage-specific specialization occurred relatively recently, in the past 20–40 My. (*b*) Common selective regimes estimated by SURFACE are indicated by symbols. The analysis was conducted using the first two PCA axes of the ten-toxin covariance matrix, but most of the convergent strategies are centered on the four major toxins. Highlighted species names and diamonds represent optima attained by many species via multiple convergent shifts. Circles represent convergent optima due to single shifts. Symbols are colored based on the toxin axes the estimated optima lie on (fig. 2 and supplementary fig. S12, Supplementary Material online). (*c*) Tiles represent the relative abundance of venom toxin in extant snakes. The overall trend is that starting from a relatively undifferentiated ancestor, snakes have increasingly focused on specific toxin families, occasionally investing in new toxin categories for their arsenals (e.g., PLA2s and SVSPs).

The clustering of distantly related species in the PCA despite the generally high phylogenetic inertia hinted at the likely parallelism of envenomation strategies across snakes. We use parallelism rather than convergence because parallelism describes a shared molecular basis, where phenotypes arise by using the same molecular mechanisms (Rosenblum et al. 2014). Thus, for our study, parallelism is a type of convergence brought about by a shared molecular basis, and we use both the terms interchangeably. Also, since our data consist of gene families shared by all the snakes, describing convergence in terms of a shared genetic structure seemed appropriate. To test for parallelism across the phylogeny, we used SURFACE (Ingram and Mahler 2013), which fits a series of stabilizing selection models to identify instances where multiple lineages adopt the same selective regime (Ingram and Mahler 2013). Our goal was to test whether macroevolutionary models involving convergent shifts to optima on a Simpsonian landscape can explain the clustering, and similarities in the venom phenotype. We do not test whether the presence or absence of a toxin family is due to convergence. SURFACE uses AIC as criterion to determine goodness of fit and keeps adding models until the AIC does not improve further (Ingram and Mahler 2013). SURFACE provides two measures of convergence: Δ*k* and *c*, which represent refinement of the adaptive landscape due to convergence, and shifts toward convergent regimes occupied by multiple lineages respectively. Although both these measures represent convergence, we use shifts to convergent regime (*c*) to classify convergence, but report both. The final model included nine regime shifts and three distinct regimes (Δ*k* = 3) and a *c = *6 convergent shifts. The AIC improved from 298.4 to 229.5 in the forward phase, to a final AIC of 211.38 in the backward phase (S11) which indicated that the final model was a better fit than the initial ones. The SURFACE model revealed widespread convergent shifts as a result of optima (the software considers parallelism and convergence to be one in the same) in elapids, vipers, and colubrids (fig. 3). Vipers showed evidence of two distinct optima, one focussed on SVMP and another on a combination of SVMP, SVSP, and PLA2 (fig. 3 and supplementary fig. 12, Supplementary Material online). One of these regimes evolved in parallel due to multiple shifts toward an optima (highlighted species names in fig. 3 and supplementary fig. 12, Supplementary Material online). The other regime focused on SVMP represents an optima in both viperids and colubrids (fig. 3) that has been achieved not due to multiple shifts but likely due to consistent use of SVMP throughout their evolutionary history. In elapids, there was greater evidence for a single convergent regime focused around TFTx that was reached by multiple shifts (fig. 3). *Pseudonaja textilis* and *Pantherophis guttatus* were the only species to have converged toward an optima focused around PLA2 via multiple shifts (fig. 3 and supplementary fig. 12, Supplementary Material online).

We used the inbuilt simulation function in SURFACE to obtain a null distribution on a simulated data set using a Hansen model that lacked true convergence (Ingram and Mahler 2013). Comparison to the null model simulations (supplementary table 2, Supplementary Material online) revealed significantly more convergent regimes (*c*) obtained from our analysis than would be obtained by chance (*p*_c_ = 0.038). This allowed us to reject the null hypothesis and conclude that species cluster together due to convergence toward some optima in the phenotypic adaptive landscape.

### Strategies Based on Major Components Evolved at Different Times

Understanding the ancestral state of a trait can paint a picture of the journey taken by the trait through evolution. We used ancestral state reconstruction (ASR) analysis to estimate recruitment times of the major venom components into the venom arsenal, and how venoms have changed throughout the course of evolution. Because of the diversity and plasticity of the venom phenotype, confidence intervals at the root were very large, and the inference of the venom in the most recent common ancestor should be interpreted with caution, particularly concerning absence of individual toxin families. Of the four major toxins that are responsible for venom diversification, the ASR detected only SVMP in the most common ancestor of the snakes (∼60 Ma, henceforth referred to as “the ancestral venom”) (fig. 3). The ASR reveals SVMP to be a major and widespread component for most of the evolutionary history of snakes. However, at the base of elapid radiation, SVMP was largely replaced by TFTx as the major component in elapid venoms. TFTx was likely present prior to the split of colubrids and elapids, but while elapids have focused primarily on TFTx, colubrids employed a combination of TFTx and SVMP throughout their evolution (McGivern et al. 2014). In vipers, SVMP has taken various paths, from being the predominant component in Viperinae (*Echis* and *Bitis*), to diversifying substantially in the Crotaline clade (*Protobothrops*, *Bothrops*, *Crotalus*, etc). The ASR suggests that high levels of PLA2 and SVSP (which is mostly restricted to vipers) are more recent additions to the venom. Although not shown in our analysis, PLA2 (both group I and group II) was most likely present at the common ancestors of both Elapids and Crotalids (Dowell et al. 2016), but became substantial parts of the venom from around 20 Ma in both these taxa as observed from their increased occurrence. Although we had estimated ancestral states for the other six components as well (supplementary figs. 23–33, Supplementary Material online), we limited our discussion to only the major toxin families since they dominate adaptive optima in the venom phylomorphospace.

## Discussion

We set out to understand how changes in gene expression underlie the evolution of a complex trait, the snake venom. First, we examined the dimensionality of this trait by estimating phylogenetic covariances between expression levels of individual toxin families. The covariances between toxin expression levels can be viewed as constraints that limit the evolution of a trait, analogously to the *G* matrix in quantitative genetics. Unlike the *G* matrix, which arises largely from pleiotropic interactions between genes, phylogenetic constraints may additionally include ecological, developmental, physiological, and other factors. Significant covariance between individual components would reflect constraints on evolutionary change and the total phenotypic space attainable by selection (Pavličev and Cheverud 2015). Thus, traits that are constituted by genes under high constraint would not be able to diversify as freely as traits with no constraint. Genetic constraints also determine convergence and parallel evolution, where high constraint reduces the likelihood of genes contributing to different convergent regimes (Rosenblum et al. 2014). Yet, for snake venom genes, we see no evidence for such constraints in gene expression, suggesting that all toxin combinations, in principle, are possible (fig. 1).

Although the lack of constraint between components implies that venom has the potential to diversify freely and fully fill the possible phenotypic space, this is far from what we observe. Rather, the total phenotypic space has surprisingly low dimensionality, with two principal components explaining 74% of the variance. Venoms form three distinct clusters around the major toxin components in the phylomorphospace, indicating the possible presence of distinct adaptive optima focussed around these toxins (figs. 2 and 3). Although snakes cluster around the major toxin components, this does not diminish the utility of the other minor components which likely impart a more nuanced and refined mode of action to the venom. However, since most species have not yet evolved the lineage specific minor components, their role in the long-term evolution of the snake venom phenotype is limited. Similar toxin-specific strategies have been observed between populations of snakes, but we show that the trend extends phylogenetically to different species as well as different families (Calvete 2017; Strickland et al. 2018). Although individual venom components do exhibit significant phylogenetic inertia (fig. 1), the phylomorphospace clusters often include unrelated taxa, suggesting shifts in envenomation strategies between adaptive optima. These shifts likely result from parallelism, which may be facilitated by lack of constraints between components (fig. 3).

Is a lack of constraint surprising for a trait like snake venom? To answer this, we need to understand one of the key processes by which novel functions and variations in gene families arise—gene duplication (Ohno 1970; Lynch and Conery 2000; Fuchs et al. 2007; Xiao et al. 2008). One of the ways gene duplication can cause functional redundancy is by producing gene copies where one of the copies carries out its designated function, whereas the other copy has no active role in the biological process, thus freeing it from selective constraints (Ohno 1970; Kondrashov et al. 2002; Innan and Kondrashov 2010). This relaxed selective constraint could allow the duplicated genes to diversify freely, as long as one of the copies performs the essential function, and the presence or absence of another copy does not affect fitness. Therefore, a system that comprises many duplicated gene families would also likely have the ability to diversify freely. Snake venom fits this characteristic since it consists of gene families that have undergone varying degrees of duplications throughout their history (Oguiura et al. 2009; Margres, Bigelow, et al. 2017). We hypothesize that the lack of constraint observed between expression levels of genes encoding for snake venom could be due to the fact that snake venom comprises duplicated genes.

One of the most prevalent theories about the origins of venom composition suggests that they originated after ancestral physiological genes underwent duplication and neofunctionalization (Casewell et al. 2013). Since venom phenotypes need to be flexible and to adapt quickly, duplicated genes make ideal toxin candidates as they are under lower selective constraints (Wong and Belov 2012; McCabe and Mackessy 2015; Sunagar et al. 2016). In addition to sequence-level changes, changes in gene expression also contribute to microevolution in snake venom (Margres, Wray, et al. 2017). To get a complete picture of the evolution of the snake venom phenotype, we need to understand how microevolution (changes in gene expression over short time scales) relates to macroevolution (selection over large time scales). From our observations, we propose a model for snake venom evolution that could potentially link the two, and explain why in spite of having the potential to freely evolve, snake venom has such low dimensionality. We propose that gene duplication facilitated recruitment of physiological genes into the venom system, following which expression levels were free to respond to natural selection due to their low constraint and to potentially occupy a wide phenotypic space. The venom compositions that provided the greatest adaptive advantage due to their favorable biochemistry of envenomation is what we see in present-day species. These observed adaptive optima are dominated by the four main toxin families leading to a high degree of parallelism. This model could likely explain why snake venom, like other systems composed of duplicated genes, experience both positive and relaxed purifying selection (Persi et al. 2016; Aird et al. 2017).

### Temporal Patterns in Venom Evolution

Ancestral snake venom composition has received considerable attention, but until now the analyses have been qualitative in nature (Calvete 2017). Although the confidence intervals for ASR are large (supplementary figs. 14–33, Supplementary Material online) owing to the remarkable evolutionary lability of venom, we can nonetheless make a number of observations about the course of evolution of major components. Among the major components, the ancestral venom most likely contained only appreciable amounts of SVMP (fig. 3). This finding is consistent with previous estimates of a likely recruitment of SVMP into the venom prior to the split of vipers from their common ancestor (∼62 Ma) (Wüster et al. 2008; Casewell et al. 2011). While we could not detect PLA2, TFTx, and SVSP with confidence in the most recent common ancestor, they could have been present at lower levels in the ancestral venom, or as ancestral precursor molecules (Jin et al. 2007; Lynch 2007; Dowell et al. 2016). This is especially likely for SVSP and PLA2 given that all three families have it in their venom at some level (fig. 3).

Being present in the ancestral venom, SVMP continued to be used as a major toxin by viperids and is still the dominant toxin family in some genera (*Echis* and *Bitis*), as well as some colubrids. However, other toxin families were recruited (or increased in quantity) later in venomous snake evolution. For example, consistent with previous work that placed recruitment of TFTx before the divergence of modern elapids (Fry et al. 2003), we also show that TFTx was likely present at the node prior to the split between elapids and colubrids. At that time TFTx may have co-occurred with SVMP prior to the split of Elapids and Colubrids, perhaps as a specific strategy, one that is quite rare in present-day snakes, being found only in the colubrid brown tree snake (*Boiga irregularis*), and to an extent in the king cobra (*Ophiophagus hannah*). With the proliferation of the TFTx family, elapids have largely lost their reliance on SVMPs.

Viperid and elapid subfamilies have convergently evolved greater reliance on PLA2 toxins (group I in elapids and group II in viperids) but have diverged in venom phenospace due to the previous co-option of different major components (TFTx for elapids and SVSP for vipers). The likely presence of PLA2 (group II) gene copies at the common ancestors of Crotalids raises questions about when the complex expanded in the course of snake evolution (Dowell et al. 2016). From our analysis, we believe that the expansion started somewhere around 20–25 Ma in vipers and was already established as a substantial part of the venom before the split of *Crotalus*, and *Protobothrops* genera. In elapids, ASR does not detect the use of PLA2 before its recruitment as a major component of coral snakes (Micrurus) about 20 Ma, but it was likely present at the common ancestor of elapids and maybe even colubrids given the convergent regime experienced by *Pseudonaja textilis* and *Pantherophis guttatus*, and its presence in many extant species. Interestingly, the recruitment of the two PLA2 families by elapids and viperids occurred at roughly the same time, perhaps as a result of convergent selection driven by radiations in prey lineages, such as mammals.

The overall trend is that recruitment of major toxin families took place at different times, and has progressed along different trajectories in different lineages, with instances of both loss and heightened expression. Snakes have then shifted focus on specific toxin families, occasionally investing into new toxin categories for their arsenals (e.g., PLA2s and SVSPs). The increased concentration of specific venom components, relative to the ancestors, has most likely happened by increases in copy number of the specific gene families (Oguiura et al. 2009; Junqueira-de-Azevedo et al. 2015; Margres, Wray, et al. 2017). Interestingly, shifts in selective regimes produced parallel specialization on the same toxin family by different snakes (fig. 3), suggesting that at the level of toxin family selection generally favors specialization as opposed to diversity.

## Conclusion

The extent to which traits are constrained by their history, versus reaching their fitness optima has been a major debate in evolutionary biology. Numerous studies have relied on phylogenetic regression to estimate morphological covariation between traits while accounting for phylogenetic nonindependence (Arnqvist and Rowe 2002; Nogueira et al. 2009; Monteiro and Nogueira 2010; Meloro et al. 2011; Adams and Felice 2014). In our approach, we analyze more than one response variable simultaneously and incorporate effects on trait relationships that arise through shared ancestry using the principles behind the animal model (Hadfield 2010; Wilson et al. 2010). We show that the structure of the gene expression PCOV can give insights into how traits evolve, by providing a conceptual bridge between micro- and macro-evolutionary forces. By showing that the phenotypic space is inherently unconstrained, we are able to highlight the existence of fitness optima and explain the existence of widespread parallelism seen in snake venoms. These findings show that in the long-term snakes are able to overcome the inherent trade-off between fitness and phylogenetic constraints. Once genes underlying more traits are known in other systems, subsequent studies will show to what extent snake venoms are typical of a general evolutionary pattern.

## Materials and Methods

### Data Collection

Toxin expression data were collected from 39 publications (Supplementary Material online). Out of the 25 reported toxin families, we selected only 10 as they were the most ubiquitous toxins amongst all snakes. We restricted our data set to include components that are found in at least 50% of snakes and eliminated low-abundance toxin families (supplementary figs. 34 and 35, Supplementary Material online). Toxins levels were recorded as per publication. Toxin values reported as absolute Fragments Per Kilobase of transcript per Million (FPKM) values were converted to a percentages of total toxin transcript expression. The phylogenetic modeling and ASR were carried out using this curated data set. The toxin values were normalized for calculating the PCOV.

### Phylogenetic Tree

We used a time-calibrated tree of squamate reptiles (snakes and lizards) based on two large data sets comprising of 44 nuclear genes for 161 squamates, and a data set of 12 genes from 4,161 squamate species, both these data sets represented families and subfamilies (Wiens et al. 2012; Pyron et al. 2013; Zheng and Wiens 2016). The result was an extensive phylogeny of squamates both in terms of sampling of genes and species. Fossil-based age constraints were used in time-calibrating the tree making it ideal for studies of biogeography, diversification, and trait evolution (Zheng and Wiens 2016). All analyses were carried out using a pruned version of this tree (supplementary fig. 13, Supplementary Material online) that contained the 52 snake species for which we collected gene expression data. This pruned tree had a time at root estimated to be ∼60 Ma.

### Estimating PCOV Matrix

To familiarize the reader with the rationale behind how the model was constructed, we provide a brief introduction to the animal model and refer the reader to Kruuk et al. (2000), Garant et al. (2004), and Wilson et al. (2010) and chapter 11 of de Villemereuil and Shinichi Nakagawa (2014) for more details. The animal model in quantitative genetics is based on the concept that provided adequate knowledge about the relationships between individuals, and measures of their phenotypic traits, we can make inferences about the patterns of inheritance and evolutionary potential of traits. At its heart is the assumption that if closely related individuals, who share most of their genes, are phenotypically more similar than unrelated individuals, who share fewer genes, we can infer that genes make a significant contribution to phenotypic variance (Wilson et al. 2010). The most basic interpretation would be that phenotypic variation (*V*_P_) is a result of additive genetic variation (*V*_A_) and a residual variance from environmental effects (*V*_R_), where the additive genetic variance (*V*_A_) is the independent effect of inherited alleles on the phenotype.

(1) $$V_{P}=V_{A}+V_{R}.$$

The partitioning of variance can also be done for multiple, covarying traits where the phenotypic covariance (COV_P_) would be the sum of additive genetic covariance (COV_A_) and covariance of residuals (COV_R_). In the animal model, “breeding value” is used as an explanatory variable for a phenotypic trait such that

(2) $$y_{i}=\mu +a_{i}+e_{i},$$

where *y_i_* is our phenotypic trait of interest, *μ* is the population mean, *a_i_* is the breeding value, and *e_i_* is the residual error. Although *a_i_* is used as an explanatory variable, its actual value is unknown and thus cannot be used to fit the model. To overcome this, we can specify the above model as a mixed effects model, with *a_i_* being modeled as random effect (Galwey 2014). By incorporating a random effect based on the pedigree of individuals, we can get an estimate of among-individual variance for the phenotypic trait (*y*) in the population. This allows us to obtain an estimate of among-individual variance in breeding values, which is defined as the additive genetic variance (*V*_A_) (Wilson et al. 2010). Therefore, the key concept behind the statistical interpretation of the animal model is that: population pedigree structure provides insights into how breeding values should covary among individuals, allowing us to solve genetic parameters like *V*_A_, and in multivariate models, COV_A_. For *n* individuals in a pedigree, the matrix of additive genetic covariance of a trait is given as *AV*_A_ where *A* is an *n* × *n* additive genetic relationship matrix containing pairwise values of relatedness. The phylogenetic linear mixed model is exactly the same as the animal model, except that instead of using a pedigree we use a phylogeny to infer additive phylogenetic covariances.

For a simple univariate trait thinking in terms of variance is sufficient, however, for multivariate models it is useful to think in terms of variance–covariance matrices. Thus, for a bivariate model of say trait 1 and trait 2, the phenotypic matrix *P* would comprise of variances for both trait 1 and trait 2 along the diagonal (*V*_P1_, *V*_P2_) and covariance between the traits (COV_P12_) such that *P* = *G* + *R*, where *G* is the additive genetic covariance matrix (or in our case the phylogenetic covariance [PCOV]), and *R* is the residual matrix. Our model was similar to model (2) and written based on the description given in section 3 on the MCMCglmm vignette for modeling multiresponse traits (Hadfield 2010). The only difference is that our model is a multivariate model with the ten toxins as response variable (*y*).

Although the genetic (or phylogenetic) effect has the potential to explain a substantial amount of phenotypic similarity, in actuality, a number of intrinsic and extrinsic variables may also be responsible. If there is speculation that such variables are important, they may be added to the model as fixed effects. This would allow us to interpret the resultant variance as having been conditioned on the specific fixed effect. However, if the additional explanatory variables are not associated with the pedigree (phylogeny in our case) then their inclusion would not alter the estimate of genetic (or phylogenetic) effect (Wilson et al. 2010). In our study, we obtained data from various studies that employ different sequencing technologies and protocols. But, since sequencing technology does not influence the phylogeny of the species, we believe that there would be no substantial change to the PCOV. For the sake of statistical fidelity however, we included sequencing technology, as reported by each study, as a fixed effect and found that there was no change to the overall PCOV structure which still largely consisted of insignificant values (Supplementary Material online).

Phylogenetic generalized linear mixed models allow for testing slightly complicated models, provide more than a simple qualitative estimate of the existence of phylogenetic structure, and have greater statistical power than typically used metric randomization approaches (Ives and Helmus 2011). The MCMC was run for a total of 20 million iterations, with burnin and thinning values of 1 million and 1,500, respectively. Diagnostics for the MCMC run were done by obtaining the plot for the MCMC and autocorrelation. The phylogenetic signal was obtained by dividing the covariance for each toxin by the total covariance of the toxin and the residuals, as mentioned in de Villemereuil and Shinichi Nakagawa (2014). More details regarding passing of fixed and random effect can be found in the Supplementary Material online. We performed principal components analysis using the phylogenetic covariances obtained from the MCMCglmm analysis. Species codes are provided in supplementary note 1, Supplementary Material online.

### Analysis of Parallelism

We used the default Ornstein–Uhlenbeck process, a convenient representation of evolution toward adaptive peaks for modeling parallelism in the SURFACE analysis (Ingram and Mahler 2013). The SURFACE method considers parallelism and convergence to be one in the same, and uses Hansen’s approach (Hansen model) of modeling evolution toward different adaptive optima by painting multiple adaptive hypothesis onto branches of a phylogenetic tree (Hansen 1997; Ingram and Mahler 2013). SURFACE is unique because unlike previous methods that utilize Hansen models, the placements of regime shifts is guided by trait data as opposed to some a priori hypothesis regarding the location of convergence (Ingram and Mahler 2013). The SURFACE method is divided into two phases. The forward phase adds successive regimes to a basic Hansen model using input from continuous trait measurements, which in our study were the first two principal components estimated from the PCOV. Using principal components from the PCOV allows us to incorporate phylogenetic effect in estimation of an adaptive landscape comprising all ten toxins in our analysis, and because the principal component axes are orthogonal, it nicely deals with the compositional nature of the data. The performance of each successive model was measured using AIC by balancing improvements in log-likelihood against increase in model complexity (Ingram and Mahler 2013). Since AIC for the models are calculated after adding log-likelihoods, the AIC for successive models may improve. The regime shift representing the best model is painted onto the tree. The backward phase is the second phase in the analysis. During this phase of SURFACE all subsets of regimes are collapsed to yield distinct regimes. The collapse is continued till the AIC of the models does not increase further. The final model has *k* regime shifts, and *k*′ distinct regimes, in addition to the extent of convergence which is defined as the difference of these terms (Δ*k*), *c* is used to represent shifts toward different convergent regimes in multiple lineages (Ingram and Mahler 2013). We used all standard parameters as mentioned in the SURFACE vignette. To obtain a null distribution, we ran 500 iterations of the inbuilt *surfaceSimulate* function using a Hansen-fit model and concatenated the output from each iteration.

### Ancestral State Reconstruction

The default parameters for the *fastAnc* function implemented in the Phytools package was used to perform the ASR (Revell 2012). *fastAnc* performs a maximum likelihood–based reconstruction by computing the root value using Felsenstein 1985 contrasts algorithm (Revell 2012). A phenogram, which shows relative positions of species in evolutionary phenospace, was plotted for each toxin using a spread cost of 0.1 (supplementary figs. 14–32, Supplementary Material online). We used the *contMap* function in Phytools to obtain a tree for changing trait values on a continuous scale represented by a color spectrum. Confidence intervals were plotted on the nodes as bars. Only traits whose confidence intervals did not overlap zero (only positive values) were considered to be present at the root. Pie charts in the main figure were drawn by calculating the relative levels of each of the major toxins estimated by the ASR at the specific node. The ancestral states for each toxin was estimated separately, and thus could not capture any (unlikely) constraint between toxin families that might have been present in the past. The ancestral states were clubbed together to only give a representative picture of what venom configuration at a particular node might have looked like. Two images in the main were obtained from Wikimedia under the creative commons license (Elapidiae: Thomas Jaehnel and Colubridae: Carlo Catoni) image for Viperidae provided by Alexander S. Mikheyev.

## Supplementary Material

Supplementary data are available at *Molecular Biology and Evolution* online.

## Supplementary Material

msz125_Supplementary_Data
